# Dynamic Quantitative Trait Locus Analysis of Seed Vigor at Three Maturity Stages in Rice

**DOI:** 10.1371/journal.pone.0115732

**Published:** 2014-12-23

**Authors:** Liangfeng Liu, Yanyan Lai, Jinping Cheng, Ling Wang, Wenli Du, Zhoufei Wang, Hongsheng Zhang

**Affiliations:** The Laboratory of Seed Science and Technology, State Key Laboratory of Crop Genetics and Germplasm Enhancement, Nanjing Agricultural University, Nanjing Jiangsu, PR China; Pennsylvania State University, United States of America

## Abstract

Seed vigor is an important characteristic of seed quality. In this study, one rice population of recombinant inbred lines (RILs) was used to determine the genetic characteristics of seed vigor, including the germination potential, germination rate, germination index and time for 50% of germination, at 4 (early), 5 (middle) and 6 weeks (late) after heading in two years. A total of 24 additive and 9 epistatic quantitative trait loci (QTL) for seed vigor were identified using QTL Cartographer and QTLNetwork program respectively in 2012; while 32 simple sequence repeat (SSR) markers associated with seed vigor were detected using bulked segregant analysis (BSA) in 2013. The additive, epistatic and QTL × development interaction effects regulated the dry maturity developmental process to improve seed vigor in rice. The phenotypic variation explained by each additive, epistatic QTL and QTL × development interaction ranged from 5.86 to 40.67%, 4.64 to 11.28% and 0.01 to 1.17%, respectively. The QTLs were rarely co-localized among the different maturity stages; more QTLs were expressed at the early maturity stage followed by the late and middle stages. Twenty additive QTLs were stably expressed in two years which might play important roles in establishment of seed vigor in different environments. By comparing chromosomal positions of these stably expressed additive QTLs with those previously identified, the regions of QTL for seed vigor are likely to coincide with QTL for grain size, low temperature germinability and seed dormancy; while 5 additive QTL might represent novel genes. Using four selected RILs, three cross combinations of seed vigor for the development of RIL populations were predicted; 19 elite alleles could be pyramided by each combination.

## Introduction

Seed vigor is an important characteristic of seed quality, reflecting potential seed germination, seedling growth, seed longevity, and tolerance to adversity [1]. Rice (*Oryza sativa* L.) is one of the most important crops in the world. Recently, improving rice vigor become more important, because the direct seeding method has become increasingly important in many Asian countries due to its lower cost and its operational simplicity [2]–[3]. For direct seeding method, the rice variety with strong seed vigor may significantly improve the speed and uniformity of seed germination, and lead to perfect field emergence of vigorous seedlings thereby leads to high competition against weeds, uniform harvest and even high yield under different conditions [4]–[5]. Seed vigor could be improved by seed treatments after harvested, such as soaking, priming, coating and infusing with the organic solvents [6]–[8]. In recent years, the emphasis on improving seed vigor has shifted to the potential of genetic improvement in breeding programs [9]. However, it is difficult to develop elite varieties with a high level of seed vigor due to a lack of understanding the mechanisms of seed vigor establishment during seed developmental stage.

Seed development is a crucial process in the lifecycle of plant, which can be divided into the two stages morphogenesis and maturation [10]. Seed vigor is affected by seed developmental period, and it has been found to be highly dependent on the stage of seed maturity [11]. Processes occurring in maturation affect seed size, oil production and protein content, as well as seedling vigor following imbibition of the dry seed for subsequent plant growth [10]. In *Arabidopsis*, the transcription factors, such as ABI3, FUS3, LEC2 and LEC1, have been identified as being master regulators of seed maturation [12]. A number of transcriptomic and proteomic studies have been undertaken to investigate the genetic program on the acquisition of desiccation tolerance during seed maturation in *Medicago trunculata* and *Arabidopsis* [13]. However, the genetic control of seed vigor establishment in the dry maturity developmental process is still unclear in rice.

Seed vigor has been known as a complex quantitative trait which makes the genetic analysis of seed vigor very difficult [5]. In the last decade, the quantitative trait loci (QTL) analysis has provided powerful tools to investigate the inheritance of seed vigor. The level of seed vigor is strongly affected by the maturity degree in rice. To date the QTL analysis was mainly focused on seed filling, grain size and yield during seed developmental stages in rice [14]–[15]. The most of previous QTL analysis of seed vigor, such as seed germination, seedling growth, seed longevity and tolerance to stress, have been conducted only at the final seed maturity developmental stage in rice [3], [5], [9], [16]–[17]. The accurate genetic regulations between seed vigor and seed maturity level are not understood.

According to the theory of developmental genetics, genes are expressed selectively at different growth stages [18]. To understand the genetic control of seed vigor establishment during seed developmental stages in rice, it is necessary to conduct dynamic QTL analysis of seed vigor during different maturity developmental stages. Epistasis, an additive-by-additive interaction between QTLs, has been recognized as contributing to the genetic control of quantitative traits [9], [19]–[22]. Seed vigor has been known as a comprehensive characteristic affected by genetic and environmental factors during seed development [1]. Besides the additive QTL, the role of epistasis QTL and the QTL × development interactions for seed vigor need to been studied in rice. However, few studies have concerned the detection of epistasis QTL and the QTL × development interactions on seed vigor establishment during maturity stages in rice.

What happens in the dry maturity developmental process to improve rice vigor is still an open question. In this study, the objectives were to investigate the genetic control of developmental behavior of seed vigor in rice. One RIL population (F_2∶10_) derived from the cross of *indica* rice IR26 and *japonica* Jiucaiqing was employed to map the loci underlying four seed vigor traits, including germination potential, germination rate, germination index and time for 50% of germination. The QTLs with additive, epistatic and QTL × development interaction effects for seed vigor were conducted during three maturity stages in rice. Finally, the novel parental combinations for seed vigor were predicted in future rice breeding. The selected RILs and identified QTLs might be used to improve seed vigor by marker assisted selection approach.

## Materials and Methods

### Plant materials and field experiment

Two rice (*Oryza sativa* L.) varieties, Jiucaiqing (*japonica*) and IR26 (*indica*), and their 150 recombinant inbred lines (RILs) (F_2∶10_) were used in this study. The 25-day-old seedlings were transplanted into a paddy field at the Experimental Station of Nanjing Agricultural University (Jiangsu Province, China; E118°50′, N32°02′) on June 20th, 2012 and also 2013. The plants were grown with 17 cm between plants within a row and 33 cm between rows. Filed management was carried out according to the local standard methods [23]. The plants with the same heading date were selected when the leading panicle emerged from the leaf sheath, and the days to heading of each RIL were estimated [22]. The main panicles of each line were harvested at the early (4 weeks after heading), middle (5 weeks after heading) and late (6 weeks after heading) maturity stages, and the harvested seeds were dried at 42°C for 7 d to a ∼13.5% seed moisture content then stored in room temperature (∼25°C) for 3 months to break seed dormancy.

### Evaluation of seed vigor

Seed germination was conducted according to the methods of Wang et al. [3], [5] with minor modifications. Fifty seeds were placed in a Petri dish (diameter 9 cm) with two sheets of filter paper, and 10 mL of distilled water was added. All Petri dishes were placed in an incubator at 30±1°C for 10 days with a 12-h light/12-h dark photoperiod. Seeds were considered to be germinated when their root lengths reached the seed length and the shoot length was half of the seed length [3], [5]. The germinability of the seeds was observed each day to calculate the germination percentage. The percentage of germinated seeds at 3 d was referred to as germination potential (GP) and the percentage of germinated seeds at 10 d was referred to as the final germination rate (GR). Germination index (GI) was calculated by the method of Wang et al. [5]: *GI* = ∑*Gt*/*t*, where *Gt* is the number of the germinated seeds on Day *t*. T_50_ is the time for 50% of germination. It's calculated by the curve fitting part of GERMINATOR software [24]. Three replications were conducted, and the mean value was used for data analysis.

### Data analysis

The experimental data were analyzed using the SPSS 19.0 software, and the phenotype of two parents was compared with Student's *t* test at 5% and 1% levels of probability. Heritability in the broad sense (*H_B_^2^*) was computed on the basis of the RIL population through analysis of variance using the formula: *H_B_^2^*  =  *σ_G_^2^*/(*σ_G_^2^* + *σ_e_^2^/n*), where *σ_G_^2^* is genetic variance, *σ_e_^2^* is error variance, and *n* is number of replicates.

### QTL mapping

The seeds harvested in 2012 were used for identification of QTLs with additive, epistatic and QTL × development interaction effects for seed vigor in rice. The genetic linkage map based on 135 simple sequence repeat (SSR) markers at an average interval of 16.5 cM was constructed by Wang et al. [22]. Using single-maturity phenotypic values, the additive QTLs were identified by the method of composite interval mapping (CIM) in QTL Cartographer 2.5. The LOD score of 2.5 was used as the threshold value to declare the presence of a putative additive QTL [9]. The QTLNetwork program ver. 2.0, based on a mixed linear model [25] was used to identify additive and epistatic QTL × development interactions for seed vigor in joint analyses of three-maturity phenotypic values. The values for testing window and filtration window were set at 10 cM, respectively, and the walking speed was 1 cM. The putative QTL detection was determined at 95% confidence level [9]. The proportion of observed phenotypic variance explained by each additive and epistatic QTL and the corresponding additive effects were also estimated. The QTL nomenclature followed the suggestion of McCouch and CGSNL [26].

### Bulk segregant analysis

The seeds harvested in 2013 were used to test those QTLs detected in 2012 by the bulked segregant analysis (BSA) method. Two extreme phenotypic bulks, including the high vigor bulk with high GP, GR, GI and lower T_50_ simultaneously and low vigor bulk with low GP, GR, GI and higher T_50_ simultaneously, were selected at each maturity stage respectively, and each bulk containing DNA from 10 individuals was used for BSA. A total of 168 SSR markers that revealed polymorphisms between Jiucaiqing and IR26 were used to determine the SSR markers associated with seed vigor.

### Prediction for novel parental combination

The best cultivars with maximum phenotypic value and elite alleles might be used to design parental combinations for crop breeding [27]. According to the information of phenotypic values and alleles of detected loci among the RILs, the best three cross combinations for seed vigor were selected. Firstly, the RILs with relatively higher GP, GR, GI or lower T_50_ at three maturity stages were selected. Then, the positive alleles of the stably expressed additive QTLs among the selected RILs were analyzed. Furthermore, the plant height and grain weight of the selected RILs were observed. Finally, the best three cross combinations were predicted to improve seed vigor in rice.

## Results

### Seed vigor of parents and RIL population

The seed vigor (GP, GR, GI and T_50_) of Jiucaiqing and IR26 and their RIL population were investigated by using seeds harvested at 4 (early), 5 (middle) and 6 (late) weeks after heading, respectively (Table 1). There were significant differences of seed vigor between two parents at three maturity stages. The Jiucaiqing had significantly higher GR and GI at the early stage, while the IR26 had significantly higher GP, GI and lower T_50_ at the middle stages and significantly higher GP and lower T_50_ at the late stage. These results indicated that the Jiucaiqing had higher seed vigor at the early stage than IR26, while IR26 had higher seed vigor at the middle and late stages. Continuous distributions and transgressive segregation were observed in all four seed vigor traits among RILs population. A two-way ANOVA showed that there was significant difference of seed vigor among RILs (*P*<0.001), indicating a large amount of genetic variation in the population. The *H*_B_^2^ of GP, GR, GI and T_50_ was more than 94% at three maturity stages.

**Table 1:** Phenotypic values of seed vigor among the parents and RIL population at three maturity stages in 2012.

Stage a	Trait b	Parents c		RILs d									
		Jiuacaiqing	IR26	Mean	SD	Min	Max	Skewness	Kurtosis	*σ_G_^2^*	*σ_e_^2^*	*H_B_^2^* (%)	*P* value
4	GP	20.0±6.7	24.4±8.8	48.6	2.76	0.0	100.0	−0.15	−0.89	0.233	0.012	94.99	4.04×10^−68^
	GR	95.6±1.6*	63.3±10.0	88.2	1.90	7.8	100.0	−2.84	7.52	0.117	0.003	97.47	2.10×10^−95^
	GI	7.7±0.3*	4.6±0.9	7.8	2.21	0.6	12.5	−1.49	2.53	14.634	0.327	97.76	2.12×10^−100^
	T_50_	3.1±0.1	3.4±0.1	3.1	0.64	2.0	5.8	1.92	5.15	1.215	0.058	95.22	6.21×10^−70^
5	GP	14.4±4.2	81.7±5.0**	40.7	2.86	0.0	95.6	0.23	−1.25	0.251	0.010	95.90	6.47×10^−76^
	GR	96.7±4.7	97.8±3.1	85.3	1.92	11.1	100.0	−2.29	5.52	0.097	0.004	95.93	3.64×10^−76^
	GI	7.5±0.3	9.8±0.3**	7.2	2.13	0.5	11.7	−0.95	0.71	13.384	0.359	97.32	6.11×10^−93^
	T_50_	3.3±0.1	2.5±0.1*	3.3	0.79	2.1	7.6	2.17	7.43	1.977	0.056	97.15	9.89×10^−89^
6	GP	36.7±9.8	95.6±0.0**	51.1	2.67	0.0	97.8	−0.21	−0.94	0.216	0.013	94.20	1.35×10^−62^
	GR	100.0±0.0	98.9±1.6	89.2	1.62	8.9	100.0	−2.56	7.58	0.079	0.003	95.97	1.57×10^−76^
	GI	8.4±0.2	10.3±0.4	7.7	2.03	0.3	10.8	−1.41	2.40	11.876	0.409	96.56	8.06×10^−83^
	T_50_	3.1±0.0	2.2±0.1*	3.1	0.75	2.1	6.6	2.51	8.04	1.579	0.071	95.50	5.22×10^−72^

a Seeds were collected at 4, 5 and 6 weeks after heading stages;

b GP, Germination potential, %; GR, germination rate, %; GI, germination index; T_50_, time for 50% of germination, d;

c Mean ± SD (standard deviation);* and ** significance at the 5% and 1% level according to Student's *t* test;

d RIL sample size n = 150, replications r = 3; SD standard deviation; *σ_G_^2^*, genetic variance; *σ_e_^2^*, error variance; *H_B_^2^*, heritability (%); *P* value, the significant difference of seed vigor among RILs according to Student's *t* test.

### Additive QTLs for seed vigor

**Figure 1:**
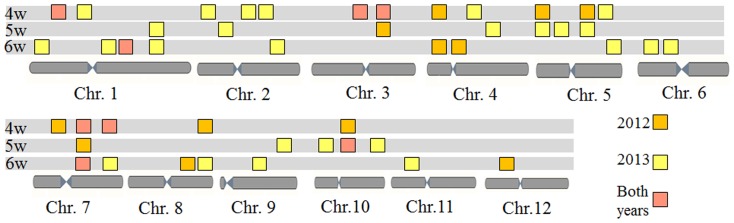
Temporal dynamics of additive QTL at 4, 5 and 6 weeks after heading in 2012 and 2013.

A total of 24 additive QTLs for seed vigor, including 5, 7, 6 and 6 QTLs for GP, GR, GI and T_50_ respectively, were identified using single-maturity phenotypic values in 2012 (Table 2). Of them, 16, 4 and 8 additive QTLs was identified at the early, middle and late maturity stages, respectively (Table 2; Fig. 1).

**Table 2:** Additive QTLs for seed vigor identified using single-maturity phenotypic values by QTL Cartographer in 2012.

Stage a	Trait b	Chr. c	QTLs	Marker Interval	LOD	A d	r^2^ (%) e
4	GP	4	*qGP4.1*	RM7585-RM335	3.27	0.10	8.88
		5	*qGP5*	RM7568-RM305	5.30	−0.12	16.31
	GR	1	*qGR1*	RM259-RM5644	3.66	−0.07	10.87
		5	*qGR5*	RM7302-RM267	2.81	−0.05	6.95
		8	*qGR8.2*	RM6976-RM6845	6.74	−0.14	30.08
		10	*qGR10*	RM5348-RM8201	2.83	−0.06	8.50
	GI	1	*qGI1.1*	RM259-RM5644	3.15	−0.72	9.20
		3	*qGI3*	RM130-RM3684	2.96	−0.78	8.10
		7	*qGI7*	RM8261-RM5426	3.79	−0.78	10.45
		8	*qGI8*	RM6976-RM6845	4.95	−1.54	29.77
		10	*qGI10*	RM5348-RM8201	3.10	−0.70	8.89
	T_50_	1	*qT_50_1.1*	RM259-RM5644	2.73	0.19	8.05
		3	*qT_50_3.1*	RM8210-RM3346	3.64	0.29	16.88
		3	*qT_50_3.2*	RM130-RM3684	4.39	0.27	11.98
		7	*qT_50_7.1*	RM1132-RM8261	3.41	0.20	9.37
		7	*qT_50_7.2*	RM6216-RM3555	2.59	0.23	13.27
5	GP	3	*qGP3*	RM130-RM3684	2.98	−0.11	10.18
	GR	7	*qGR7*	RM8261-RM5426	3.03	−0.06	9.86
	GI	7	*qGI7*	RM8261-RM5426	3.08	−0.68	9.51
	T_50_	3	*qT_50_3.2*	RM130-RM3684	3.80	0.33	11.69
6	GP	4	*qGP4.1*	RM7585-RM335	2.85	0.11	10.29
		4	*qGP4.2*	RM335-RM518	2.88	0.11	12.25
		7	*qGP7*	RM8261-RM5426	2.96	−0.09	9.93
	GR	8	*qGR8.1*	RM6215-RM342	3.14	−0.10	19.02
		12	*qGR12*	RM1880-RM20	2.73	−0.16	40.67
	GI	1	*qGI1.2*	RM6950-RM5759	2.91	0.66	10.15
		7	*qGI7*	RM8261-RM5426	3.04	−0.71	10.05
	T_50_	1	*qT_50_1.2*	RM6950-RM5759	3.86	−0.26	13.22

a Seeds were collected at 4, 5 and 6 weeks after heading;

b GP, Germination potential; GR, germination rate; GI, germination index; T_50_, time for 50% of germination;

c Chromosome on which the QTL was located;

d Additive effect is the effect of substituting an IR26 allele for a Jiucaiqing allele; Its positive value indicates that IR26 has the positive allele; The case of negative values is just the opposite;

e Variation explained by each putative QTL.

There were sixteen additive QTLs identified at the early maturity stage (Table 2), including two for GP (*qGP4.1* and *qGP5*), four for GR (*qGR1*, *qGR5*, *qGR8.2* and *qGR10*), five for GI (*qGI1.1*, *qGI3*, *qGI7*, *qGI8* and *qGI10*), and five for T_50_ (*qT_50_1.1, qT_50_3.1, qT_50_3.2, qT_50_7.1* and *qT_50_7.2*). The phenotypic variance explained by each QTL ranged from 6.95% to 30.08%. Two major QTLs *qGR8.2* and *qGI8* (r^2^>20%) were identified with a LOD score of 6.74 and 4.95 respectively, explaining 30.08% and 29.77% of the phenotypic variation, between RM6976 and RM6845 region on chromosome 8.

There were four additive QTLs, each one for GP (*qGP3*), GR (*qGR7*), GI (*qGI7*) and T_50_ (*qT_50_3.2*), identified at the middle maturity stage (Table 2). The *qGP3* and *qT_50_3.2* were co-located between RM130 and RM3684 region on chromosome 3. Similarly, the *qGR7* and *qGI7* were co-located between RM8261 and RM5426 region on chromosome 7. The phenotypic variance explained by each QTL ranged from 9.51% to 11.69%.

There were eight additive QTLs identified at the late maturity stage (Table 2), including three for GP (*qGP4.1*, *qGP4.2* and *qGP7*), two for GR (*qGR8.1* and *qGR12*), two for GI (*qGI1.2* and *qGI7*) and one for T_50_ (*qT_50_1.2*). Among these QTLs, the *qGI1.2* was co-located with *qT_50_1.2* between RM6950 and RM5759 region on chromosome 1, and the *qGI7* was co-located with *qGR7* between RM8261 and RM5426 region on chromosome 7. The phenotypic variance explained by each QTL ranged from 9.93% to 40.67%. One major QTL *qGR12* (r^2^>20%) was identified with a LOD score of 2.73, explaining 40.67% of the phenotypic variation, between RM1880 and RM20 region on chromosome 12.

### Epistatic QTL for seed vigor

A total of nine epistatic QTLs were identified in joint analysis of three-maturity phenotypic values in 2012 (Table 3). Of them, each three epistatic QTLs were identified for GR, GI and T_50_ respectively. The epistatic QTL was detected with only epistatic main effect, while no significant QTL × development interaction effect. The phenotypic variance explained by each epistatic QTL ranged from 4.64% to 11.28%, and the phenotypic variation explained by each QTL × development interaction ranged from 0.04% to 1.17%.

**Table 3:** Epistatic QTL for seed vigor identified in joint analysis of three-maturity values by QTLNetwork in 2012.

Trait a	Loci (*i*) b		Loci (*j*) b		AA c	AAD d			r^2^ (AA) (%) e	r^2^ (AAD) (%) e
	Chr.	Interval	Chr.	Interval		AAD1	AAD2	AAD3		
GR	2	RM5404-RM208	7	RM5426-RM6216	0.052**	0.0001	−0.0001	0.0000	11.28	0.42
	2	RM208-RM6312	7	RM3555-RM1306	0.048**	0.0023	−0.0007	−0.0016	9.50	0.88
	7	RM6216-RM3555	10	RM8202-RM6691	−0.044**	−0.0023	−0.0179	0.0205	4.64	1.17
GI	2	RM3264-RM5340	2	RM5427-RM5651	−1.075**	−0.0901	−0.0449	0.1384	9.84	0.73
	2	RM5404-RM208	7	RM8261-RM5426	0.450**	0.0000	0.0000	0.0000	7.20	0.15
	2	RM208-RM6312	7	RM3555-RM1306	0.512**	0.0509	−0.0336	−0.0170	7.04	0.72
T_50_	3	RM282-RM6080	7	RM1132-RM8261	0.222**	0.0001	−0.0001	0.0000	5.74	0.04
	3	RM6080-RM6959	7	RM3555-RM1306	−0.208**	0.0002	0.0000	−0.0002	5.14	0.15
	8	RM342-RM3459	12	RM1261-RM7344	0.246**	−0.0001	0.0002	−0.0001	8.35	0.29

a Seeds were collected at 4, 5 and 6 weeks after heading; GP, Germination potential; GR, germination rate; GI, germination index; T_50_, time for 50% of germination;

b Chromosome on which the QTL was located;

c AA represents the estimated additive effects of epistatic QTL; ^**^ indicates significance at the level of 1%; its positive value indicates that two loci genotypes being the same as those in parent Jiucaiqing (or IR26) take the positive effects, while the two-loci recombinants take the negative effects;

d AAD1, AAD2 and AAD3 represents the additive effect of epistatic QTL for the vigor of seeds collected at 4, 5 and 6 weeks after heading, respectively; its positive value indicates that two loci genotypes being the same as those in parent Jiucaiqing (or IR26) take the positive effects, while the two-loci recombinants take the negative effects;

e r^2^ (AA) represents the phenotypic variation explained by the epistatic QTL; r^2^ (AAD) represents the phenotypic variation explained by the epistatic QTL × development interactions.

### Additive QTL × development interactions for seed vigor

A total of five additive QTLs were identified in joint analysis of three-maturity phenotypic values in 2012 (Table 4). Each one additive QTL was identified for GP, GR and GI respectively, and two additive QTLs was identified for T_50_. The additive QTL was detected with only additive main effect, while no significant QTL × development interaction effect. The phenotypic variance explained by each additive QTL ranged from 5.86% to 9.84%, and the phenotypic variation explained by each QTL × development interaction ranged from 0.01% to 1.08%.

**Table 4:** Additive QTL × development interactions for seed vigor identified in joint analysis of three-maturity values by QTLNetwork in 2012.

Trait a	Loci b		A c	AD d			r^2^ (A) (%) e	r^2^ (AD) (%) e
	Chr.	Interval		AD1	AD2	AD3		
GP	7	RM1132-RM8261	−0.098**	0.0001	0.0001	0.0001	8.21	0.29
GR	2	RM5804-RM1920	−0.046**	0.0000	−0.0001	0.0001	5.86	0.82
GI	7	RM8261-RM5426	−0.521**	0.0000	0.0000	0.0000	9.84	0.01
T_50_	3	RM3564-RM3684	0.260**	0.0411	0.0344	−0.0750	8.33	1.08
	7	RM8261-RM5426	0.216**	0.0000	−0.0001	0.0000	7.67	0.01

a Seeds were collected at 4, 5 and 6 weeks after heading; GP, Germination potential; GR, germination rate; GI, germination index; T_50_, time for 50% of germination;

b Chromosome on which the QTL was located;

c A represents the estimated additive effects of additive QTL; ^**^ indicates significance at the level of 1%; its positive value indicates that Jiucaiqing has the positive allele and the case of negative values is just the opposite;

d AD1, AD2 and AD3 represents the additive effect of additive QTL for the vigor of seeds collected at 4, 5 and 6 weeks after heading, respectively; its positive value indicates that Jiucaiqing has the positive allele and the case of negative values is just the opposite;

e r^2^ (A) represents the phenotypic variation explained by the additive QTL; r^2^ (AD) represents the phenotypic variation explained by the additive QTL × development interactions.

### Bulk segregant analysis for seed vigor

**Figure 2:**
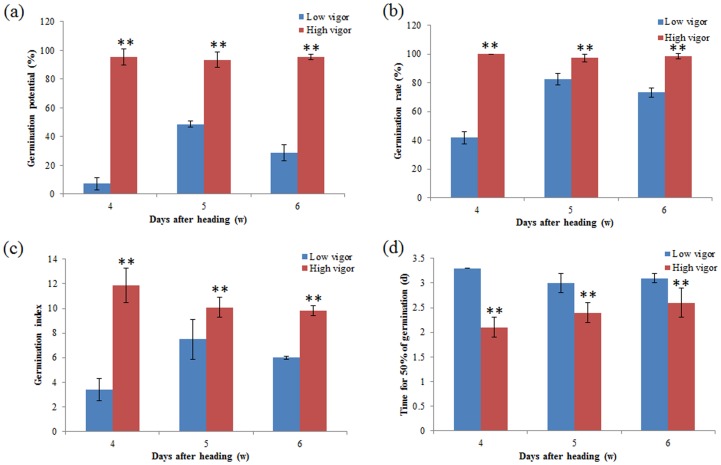
Phenotype of high and low vigor bulks in seeds collected at 4, 5 and 6 weeks after heading in 2013. (a) germination potential (%); (b) germination rate (%); (c) germination index; (d) time for 50% of germination (d); ** significance at the 1% level according to Student's *t* test.

To test those additive QTLs detected in 2012, the SSR markers associated with seed vigor were identified using BSA method in 2013. Two extreme phenotypic bulks, including the high and low vigor bulks, were selected at each maturity stage respectively (Fig. 2). A total of 32 SSR markers associated with seed vigor were identified at three maturity stages (Table 5). Of them, 11, 10 and 13 SSR markers were identified at the early, middle and late maturity stages, respectively (Fig. 1). By comparison, a total of 11 SSR markers near to the locations of the additive QTLs identified in 2012; 20 additive QTLs might be stably expressed over two years (Table 5).

**Table 5:** SSR markers associated with seed vigor identified by BSA method in 2013 and the co-located additive QTLs identified in 2012.

Stage a	Linkage marker	Chr. b	Additive QTLs identified in 2012 c	Stage a	Linkage marker	Chr. b	Additive QTLs identified in 2012 c	Stage a	Linkage marker	Chr. b	Additive QTLs identified in 2012 c
4	RM580	1	*qGI1.1 qGR1 qT_50_1.1*	5	RM3362	1		6	RM3362	1	
	RM7419	1			RM5427	2			RM84	1	
	RM5622	2			RM16626	4			RM128	1	
	RM5631	2			RM267	5	*qGR5*		RM5759	1	*qGI1.2 qT_50_1.2*
	RM5404	2			RM1366	5			RM6312	2	
	RM3346	3	*qT_50_3.1*		RM7568	5	*qGP5*		RM274	5	
	RM130	3	*qGI3 qT_50_3.2 qGP3*		RM6570	9			RM3183	6	
	RM16535	4			RM7492	10			RM6818	6	
	RM421	5			RM8201	10	*qGI10 qGR10*		RM5426	7	*qGP7*
	RM8261	7	*qGI7 qT_50_7.1 qGR7 qGP7*		RM6824	10			RM3555	7	*qT_50_7.2*
	RM3555	7	*qT_50_7.2*						RM6845	8	*qGI8 qGR8.2*
									RM7039	9	
									RM167	11	

a Seeds were collected at 4, 5 and 6 weeks after heading;

b Chromosome on which the marker associated with seed vigor was located;

c The marker associated with seed vigor was near to the location of additive QTL identified in 2012.

### Prediction for novel parental combination

**Figure 3:**
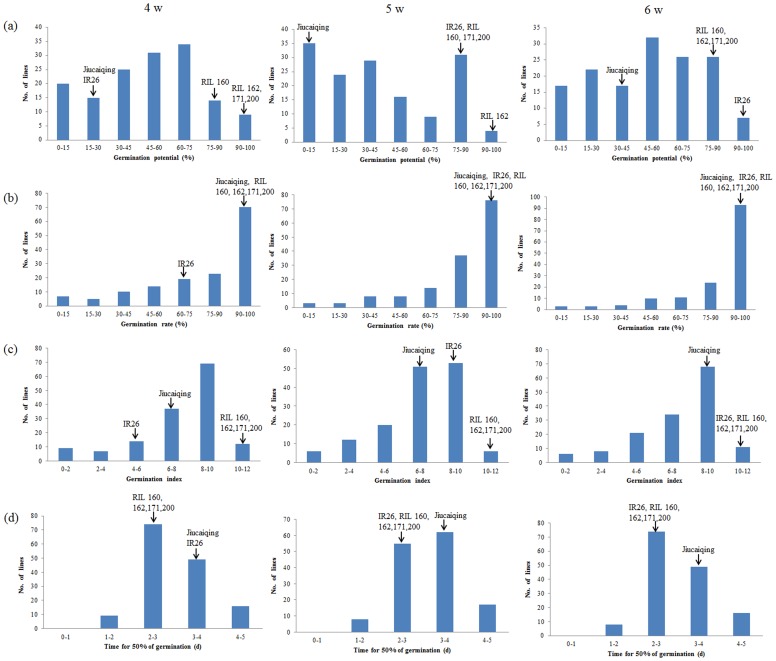
Frequency distributions of seed vigor traits among RILs and the phenotype of selected RILs in seeds collected at 4, 5 and 6 weeks after heading. (a) germination potential (%); (b) germination rate (%); (c) germination index; (d) time for 50% of germination (d).

**Figure 4:**
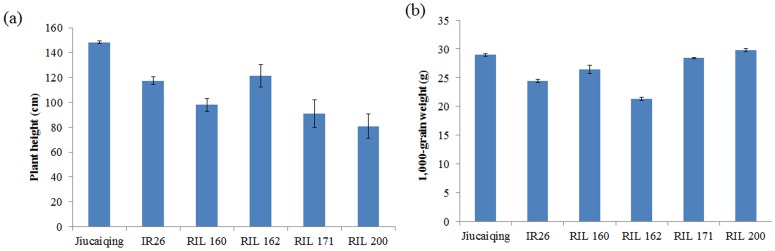
Plant height and 1,000-grain weight of Jiucaiqing and IR26 and the selected RILs. (a) plant height (cm); (b) 1,000-grain weight (g) of seeds collected at 6 weeks after heading.

According to the phenotype of the selected RILs and the positive alleles of the 20 additive QTLs which stably expressed in two years, the best cross combinations for the development of RIL populations were predicted to improve seed vigor. Four RILs were selected with a relatively higher value of GP, GR and GI, and a relatively lower value of T_50_ at three maturity stages respectively (Fig. 3). The selected RILs have 14 to 17 positive alleles of the additive QTLs (Table 6). Furthermore, the selected RILs have favorable plant height and 1,000-grain weight (Fig. 4). To improve seed vigor, the best three cross combinations (RIL 160 × RIL 162, RIL 162 × RIL 171 and RIL 162 × RIL 200) for seed vigor were predicted; 19 elite alleles could be pyramided by each combination (Table 6).

**Table 6:** The positive alleles of additive QTLs which stably expressed in two years in the selected RILs.

Traits a	QTLs b	Selected RILs c			
		160	162	171	200
GI	*qGI1.1*	+	+	+	+
	*qGI1.2*		+		
	*qGI3*	+	+	+	+
	*qGI7*	+	+	+	+
	*qGI8*	+	+		+
	*qGI10*	+	+	+	
GP	*qGP3*	+	+	+	+
	*qGP5*	+		+	+
	*qGP7*	+	+	+	+
GR	*qGR1*	+		+	+
	*qGR5*	+		+	
	*qGR7*	+	+	+	+
	*qGR8.2*	+	+		+
	*qGR10*	+	+	+	
T_50_	*qT_50_1.1*	+	+	+	+
	*qT_50_1.2*		+		
	*qT_50_3.1*			+	+
	*qT_50_3.2*	+	+	+	+
	*qT_50_7.1*	+			+
	*qT_50_7.2*	+	+		+

a GP, Germination potential; GR, germination rate; GI, germination index; T_50_, time for 50% of germination;

b The additive QTLs were detected in both years;

c + The positive allele were detected in the selected RIL.

## Discussion

Seed vigor is acquired during seed maturity. Hence, understanding the relationship between seed maturity and seed vigor is important for seed production [11]. In rice, the days from heading to harvest are 25–30 days for the early season rice and 36–44 days for the late season rice in Nanjing (Jiangsu Province, China; E118°50′, N32°02′) [23]. Therefore, the vigor of seeds harvested at 4, 5 and 6 weeks after heading was investigated respectively in this study. Dynamic analysis showed that the *indica* IR26 variety tended to have higher vigor than the *japonica* Jiucaiqing at the middle and late maturity stages. In the evaluation of seed vigor, the speed of seed germination is a desirable trait for seed vigor testing [5]; thereby the GP, GI and T_50_ were used to predict seed vigor in the present study. These parameters must be simultaneously rather than separately considered in seed quality evaluation [11]. For example, the 4 weeks after heading could be considered as a harvest period for the Jiucaiqng according to the GR reached to 95.6%; however it was not a suitable time for harvesting due to its lower GP and GI. In the future breeding, seed vigor should be considered as a selection criterion to get better hybrid variety with higher yield and quality of seeds.

In crops, the emphasis on seed production is associated with dry weight accumulation and yield, so more attention has been focused on physiological maturity, which is the point in development when dry matter accumulation is maximal [28]. In this study, the physiological maturity in IR26 and Jiucaiqing occurred at around 5 and 6 weeks after heading respectively. The seed vigor of the RIL population showed significant differences at the three maturity stages. Some genotypes can achieve high seed vigor if seed is harvested before physiological maturity, while the seed vigor of most genotypes continues to increase after physiological maturity. The final stages of maturity and drying have been considered to be important to development of seed vigor [28]. Thus, the IR26 had higher vigor than Jiucaiqing at the middle and late stages due to its earlier physiological maturity and drying after 5 weeks of heading. A large number of changes in gene expression and resultant metabolism occur in seeds during maturity and drying stages [29], including the accumulation of putatively protective molecules, e.g. late embryogenesis abundant (LEA) proteins and dehydrins, the metabolic ‘switching off’ and the presence and operation of repair systems during rehydration of seeds [28]. In this study, we try to explore the genetic control of seed vigor establishment during maturity stages.

Understanding the gene action will help plant breeders to determine the selection strategy in breeding programs. Because the performance of quantitative traits were all affected by genetic factors and multi-environment elements together, it is important to identify the true QTL in multi-environmental conditions simultaneously. Thus, the QTL identified in 2012 were tested in 2013 by the BSA method. BSA is a low cost and rapid technique to detect large effect QTL alleles in a large sample of progenies [30]–[31], which has been used to identify the markers linked to complex quantitative traits such as yield [30] and drought tolerance [32] in rice. In this study, 20 additive QTLs identified in 2012 were also detected by BSA in 2013. These co-localized loci might be stably expressed in both years. The changeful weather could affect the grain filling and seed maturity degree, then resulting to a different QTL expression at different years.

In this study, the identification of QTLs was conducted at three maturity stages (4, 5 and 6 weeks after heading), which will greatly facilitate the detection of QTLs and QTL × development interactions. By comparison, the more additive QTLs were expressed at the early maturity developmental stages followed by the late maturity stage. We found that the expressions of QTL were differential at different maturity stages: the additive QTLs rarely co-localized among the different stages. Only the *qGP4.1*, *qGI7* and *qT_50_3.2* were expressed at two maturity stages in 2012 and the marker RM3362 and RM3555 associated with seed vigor were identified at two maturity stages in 2013. These co-located QTLs might play important roles in controlling the seed vigor during different maturity stages. For the traits of seed vigor, the identification of co-localized additive QTLs occurred in the genomic RM259-RM5644 of chromosome 1 for *qGR1*, *qGI1.1* and *qT501.1*, the genomic region RM6950-RM5759 of chromosome 1 for *qGI1.2* and *qT_50_1.2*, the genomic region RM130–RM3684 of chromosome 3 for *qGP3*, *qGI3* and *qT_50_3.2*, the genomic region RM8261–RM5426 of chromosome 7 for *qGP7*, *qGR7*, *qGI7* and *qT_50_7.1*, the genomic region RM6976-RM6845 of chromosome 8 for *qGR8.*2 and *qGI8* and the genomic region RM5348-RM8201 of chromosome 10 for *qGI10* and *qGR10.* These co-localized QTLs could be very useful in the simultaneous improvement of more than one trait. Additionally, the joint analysis of the multi-maturity phenotypic values suggested that epistatic QTLs and QTL × development interactions were important components for seed vigor, even though the degree of interaction was low.

The additive QTLs which stably expressed over years might play important roles in seed vigor in different environments. Comparing the positions of these 20 stably expressed additive QTLs detected here with other QTLs reported previously, we found that the region of QTL *qGR1*, *qGI1.1* and *qT_50_1.1* on chromosome 1 coincided with *qGL-1* for grain length [33]. The region of QTL *qGI1.2* and *qT_50_1.2* on chromosome 1 was similar with the region of *qGRL1.1* for grain dimension [34] and *qSD1* for seed dormancy [22]. The QTL *qT_50_3.1* was on the similar location of *qSD3.2* for seed dormancy [23] on chromosome 3. The *qGR5* located on the same region of *qSD-5* for seed dormancy [35] and *qLTG-5-1* for seed low temperature germinability [36] on chromosome 5. In addition, the *qT_50_7.1* is near to *Sdr4* for seed dormancy [37] and *qGL7-2* for grain length [38]. The region of QTL *qGI7*, *qGR7* and *qGP7* on chromosome 7 was similar with the region of *qSD7.1* for seed dormancy [22]. The QTL *qT_50_7.2* was on the similar location of *qGL7* for grain length [39] on chromosome 7. Furthermore, *qGI8* and *qGR8.2* located on the same region of *qSD8* for seed dormancy on chromosome 8 [40], and *qGI10* and *qGR10* located on the same region of *qLTG-10* for seed low temperature germinability [36] on chromosome 10. However, there were no QTLs previously reported to be close to *qGI3*, *qT_50_3*.2, *qGP3*, *qGP4*.1 and *qGP5* which indicates that these additive QTLs might be novel genes. With the increase in the number of QTLs identified for seed vigor, the genetic control of seed vigor will be better understood.

The exploration of physiological and genetic mechanisms in seed vigor is a highlight of seed science [5]. In this study, we found the regions of QTLs for seed vigor are likely to coincide with QTLs for grain size, low temperature germinability and seed dormancy, suggesting that they are partly under the control of the same genetic mechanism. Of them, *Sdr4* has been cloned and identified as one of the major determinants of dormancy in rice, which affects the expression of several *DELAY OF GERMINATION 1-LIKE (DOG1-LIKE)* genes [37]. *Sdr4* expression is positively regulated by *OsVP1*, a global regulator of seed maturation that is orthologous to maize viviparous 1 (*VP1*) and *Arabidopsis ABI3* [37], [41]–[42]. Furthermore, we found that the region of *qGI1.2* and *qT_50_1.2* identified in this study was similar with the location of *OsVP1* on chromosome 1 [43], indicating the seed vigor is regulated by the *OsVP1* during seed maturation. To date, only one a major rice QTL *qLTG3-1* for low temperature germinability has been cloned [44]. The *qLTG3-1* is functionally associated with the vacuolation of the tissues covering the embryo, which results in the reduction of the mechanical resistance to coleoptile growth. In this study, one major QTLs *qGR8.2* was co-located with *qSD8* for seed dormancy [40]. Fine mapping the *qGR8.2* is now in progress to elucidate the molecular mechanism of seed vigor using near isogenic lines (NILs).

Besides gene functional analysis, the genetic mechanisms of seed vigor can also be well revealed by functional mapping. Functional mapping integrates developmental principle of trait formation into a QTL framework, which can detect the specific QTLs that determine the developmental pattern of a complex trait [45]–[52]. Within the framework of functional mapping, we can understand that when is a QTL switched on to affect seed vigor and how long will the genetic effect of the QTL last [52]. Recently, functional mapping, merged with the idea of quantitative trait nucleotides (QTNs) mapping, led to the identification of specific sequence variants that underlie developmental changes [52]. In this study, the traditional interval mapping was used; to enhance the understanding of the genetic control of developmental changes of seed vigor, the functional mapping could be used in further study.

In a hypothetical cross of two cultivars, the trait values of produced RILs can be predicted by the effects of all the detected loci. The best RIL with maximum value would represent the best cross. To improve seed vigor, all the elite alleles might be pyramided into one cultivar as far as possible. Thus, according to the information of phenotypic values and the allelic effects of the 20 stably expressed additive QTLs in two years, the best three cross combinations for the development of RIL populations were predicted in this study. A total of 19 elite alleles could be pyramided by each combination to improve seed vigor. These results showed that the selected RILs as new materials will be valuable in future rice breeding programs. The identified QTLs will be applicable to improve rice seed vigor by marker assisted selection.
